# Supplementary material for: Hypertension subtypes at high altitude in Peru: Analysis of the Demographic and Family Health Survey 2016–2019
Source: PLoS One. 2024 Apr 12;19(4):e0300457. doi: 10.1371/journal.pone.0300457 (PMC11014732; doi:10.1371/journal.pone.0300457)
Supplement: S1 File — (PDF) [file pone.0300457.s001.pdf]

S1 Table. General characteristics of participants according to hypertension (N = 122,336).

perf	Without Hypertension<br>% (95% CI)	Hypertension<br>% (95% CI)	*p*
Age			<0.001
18-44	91.5 (91.2-91.9)	8.5 (8.1-8.8)	
45-54	76.1 (75.0-77.1)	23.9 (22.9-25.0)	
55-64	63.7 (62.4-65.0)	36.3 (35.0-37.6)	
65-74	48.7 (47.0-50.4)	51.3 (49.6-53.0)	
>75	38.4 (36.3-40.6)	61.6 (59.4-63.7)	
Sex			<0.001
Female	81.4 (80.9-82.0)	18.6 (18.0-19.1)	
Male	78.0 (77.3-78.6)	22.0 (21.4-22.7)	
Area of residence			<0.001
Urban	79.0 (78.5-79.5)	21.0 (20.5-21.5)	
Rural	82.3 (81.7-82.8)	17.7 (17.2-18.3)	
Level of completed education			<0.001
No formal education	66.4 (65.5-67.3)	33.6 (32.7-34.5)	

Primary	83.1 (82.3-84.0)	16.9 (16.0-17.7)	
Secondary	83.2 (82.5-83.8)	16.8 (16.2-17.5)	
Higher	83.7 (83.0-84.3)	16.3 (15.7-17.0)	
Household wealth index			<0.001
Poorest	82.0 (81.4-82.6)	18.0 (17.4-18.6)	
Poor	82.5 (81.8-83.1)	17.5 (16.9-18.2)	
Middle	80.6 (79.8-81.4)	19.4 (18.6-20.2)	
Wealthy	77.8 (76.8-78.8)	22.2 (21.2-23.2)	
Wealthiest	75.5 (74.3-76.6)	24.5 (23.4-25.7)	
BMI			<0.001
Underweight / Normal	86.6 (86.0-87.1)	13.4 (12.9-14.0)	
Overweight	79.9 (79.3-80.5)	20.1 (19.5-20.7)	
Obesity	69.6 (68.6-70.5)	30.4 (29.5-31.4)	
Daily smoking status	74.2 (70.8-77.4)	25.8 (22.6-29.2)	<0.001
Self-reported diabetes	46.5 (43.9-49.1)	53.5 (50.9-56.1)	<0.001
Altitude			<0.001
Low altitude	78.8 (78.3-79.3)	21.2 (20.7-21.7)	
High altitude	83.0 (82.4-83.6)	17.0 (16.4-17.6)	

CI: Confidence interval; BMI: Body Mass Index.

S2 Table. Association between high altitude and hypertension (avoiding the white coat effect) (N = 110,304).

	Crude Model			Adjusted Model †		
	**cPR**	**95% CI**	***p***	**aPR**	**95% CI**	***p***
Altitude						
0-2,500 masl	Ref.			Ref.		
≥2,500 masl	0.81	0.78-0.85	<0.001	0.90	0.86-0.94	<0.001

cPR: crude Prevalence Ratio, aPR: adjusted Prevalence Ratio, CI: Confidence Interval, masl: meters above sea level; Ref.: Reference.

† Model adjusted for: age, sex, BMI, daily smoker status, self-reported diabetes, area of residence, level of completed education, household wealth index.

Table 3S. Association between high altitude and hypertension subtypes (avoiding white coat effect) (N = 100,881).

	ISH				IDH				SDH			
	cRPR	95% CI	aRPR	95% CI	cRPR	95% CI	aRPR	95% CI	cRPR	95% CI	aRPR	95% CI
**In all population (N = 100,881)**												
0-2,500 masl	Ref.		Ref.		Ref.		Ref.		Ref.		Ref.	
≥2,500 masl	0.74	0.67-0.81	**0.67**	**0.60-0.74**	1.03	0.86-1.24	**1.57**	**1.30-1.90**	0.79	0.69-0.92	1.02	0.87-1.19

aRPR: adjusted relative prevalence ratio; cRPR: crude relative prevalence ratio; CI: Confidence Interval, masl: meters above sea level; ISH: Isolated Systolic Hypertension; IDH: Isolated Diastolic Hypertension: SDH: Systolic-diastolic Hypertension.

Model adjusted for: age, sex, BMI, daily smoker status, self-reported diabetes, area of residence, level of completed education, household wealth index.

Table 4S. Sensitivity analysis of the association between hypertension (mean of both measurements) and altitude (0-500-2500-3500 masl) (N = 122,336).

	**Crude Model**			**Adjusted Model †**		
	**cPR**	**95% CI**	***p***	**aPR**	**95% CI**	***p***
Altitude						
<500 masl	Ref.			Ref.		
500-2,499 masl	0.88	0.84-0.92	<0.001	0.94	0.90-0.98	0.002
2,500-3,499 masl	0.79	0.75-0.83	<0.001	0.88	0.84-0.92	<0.001
≥3,500 masl	0.77	0.72-0.82	<0.001	0.86	0.81-0.92	<0.001

cPR: crude Prevalence Ratio, aPR: adjusted Prevalence Ratio, CI: Confidence Interval, masl: meters above sea level; Ref.: Reference.

† Model adjusted for: age, sex, BMI, daily smoker status, self-reported diabetes, area of residence, level of completed education, household wealth index.

Table 5S. Sensitivity analysis of the association between hypertension subtypes (average of both measurements) and altitude. (0-500-2,500-3,500) (N = 111 558).

	**ISH**				**IDH**				**SDH**			
	cRPR	95% CI	aRPR	95% CI	cRPR	95% CI	aRPR	95% CI	cRPR	95% CI	aRPR	95% CI
**In all population (N = 111,558)**												
<500 masl	Ref.		Ref.		Ref.		Ref.		Ref.		Ref.	
500-2,499 masl	0.80	0.72-0.88	**0.77**	**0.69-0.85**	1.10	0.85-1.40	**1.36**	**1.06-1.75**	0.73	0.63-0.85	0.83	0.71-0.97
2,500-3,499 masl	0.72	0.65-0.80	**0.65**	**0.58-0.73**	1.12	0.91-1.38	**1.70**	**1.37-2.12**	0.72	0.62-0.84	0.93	0.80-1.10
≥3,500 masl	0.67	0.59-0.75	**0.55**	**0.47-0.63**	1.06	0.81-1.39	**1.84**	**1.37-2.47**	0.69	0.56-0.84	0.92	0.74-1.13

aRPR: adjusted relative prevalence ratio; cRPR: crude relative prevalence ratio; CI: Confidence Interval, masl: meters above sea level; ISH: Isolated Systolic Hypertension; IDH: Isolated Diastolic Hypertension: SDH: Systolic-diastolic Hypertension.

Model adjusted for: age, sex, BMI, daily smoker status, self-reported diabetes, area of residence, level of completed education, household wealth index.

Table 6S. Sensitivity analysis of the association between hypertension (avoiding white coat effect) and altitude (0-500-2500-3500 masl) (N = 110 304).

	**Crude Model**			**Adjusted Model †**		
	**cPR**	**95% CI**	***p***	**aPR**	**95% CI**	***p***
Altitude						
<500 masl	Ref.			Ref.		
500-2,499 masl	0.89	0.84-0.93	<0.001	0.95	0.91-0.99	0.033
2,500-3,499 masl	0.80	0.75-0.84	<0.001	0.89	0.84-0.94	<0.001
≥3,500 masl	0.79	0.74-0.85	<0.001	0.88	0.82-0.95	0.001

cPR: crude Prevalence Ratio, aPR: adjusted Prevalence Ratio, CI: Confidence Interval, masl: meters above sea level; Ref.: Reference.

† Model adjusted for: age, sex, BMI, daily smoker status, self-reported diabetes, area of residence, level of completed education, household

Table 7S. Sensitivity analysis of the association between hypertension subtypes (avoiding white coat effect) and altitude (0-500-2,500-3,500) (N = 100,881).

	**ISH**				**IDH**				**SDH**			
	cRPR	95% CI	aRPR	95% CI	cRPR	95% CI	aRPR	95% CI	cRPR	95% CI	aRPR	95% CI
**In all population (N = 110,304)**												
<500 masl	Ref.		Ref.		Ref.		Ref.		Ref.		Ref.	
500-2,499 masl	0.80	0.72-0.90	**0.77**	**0.69-0.86**	1.05	0.84-1.30	**1.33**	**1.06-1.66**	0.76	0.64-0.90	0.86	0.72-1.03
2,500-3,499 masl	0.72	0.65-0.81	**0.64**	**0.57-0.73**	1.10	0.88-1.38	**1.71**	**1.36-2.16**	0.75	0.63-0.89	0.95	0.79-1.15
≥3,500 masl	0.68	0.59-0.78	**0.55**	**0.47-0.64**	0.94	0.71-1.23	**1.66**	**1.24-2.23**	0.77	0.61-0.97	1.02	0.81-1.30

aRPR: adjusted relative prevalence ratio; cRPR: crude relative prevalence ratio; CI: Confidence Interval, masl: meters above sea level; ISH: Isolated Systolic Hypertension; IDH: Isolated Diastolic Hypertension: SDH: Systolic-diastolic Hypertension.

Model adjusted for: age, sex, BMI, daily smoker status, self-reported diabetes, area of residence, level of completed education, household wealth index.
