# Supplementary material for: Hypertension subtypes at high altitude in Peru: Analysis of the Demographic and Family Health Survey 2016–2019
Source: PLoS One. 2024 Apr 12;19(4):e0300457. doi: 10.1371/journal.pone.0300457 (PMC11014732; doi:10.1371/journal.pone.0300457)
Supplement: Attachment — Submitted filename: Review Plos Paper.docx [file pone.0300457.s002.docx]

Review:-

I appreciate the opportunity to review this interesting study “Hypertension subtypes at high altitude in Peru. Analysis of demographic and family health survey 2016-2019.”

Here is my few suggestion for consideration:-

**Introduction section:**- While few references have been mentioned regarding status of hypertension in high altitude regions of the world like south America, Nepal and Tibet, conspicuously missing is the reference on this subject in the Trans-Himalayan high altitude population of Indian part of Himalaya in Ladakh one of the highest inhabited regions in the Northernmost part of India. (BMJ Open 2015.) Reference 1. This study mentioned above concludes that like everywhere else in the world, hypertension prevalence in a high altitude population has multifactorial etiology. It shows that age, gender, socio-economic factors, culture, race and changing life style play a big role with the effect of high altitude itself on the prevalence of hypertension.

**Method section:-**

Being a secondary cross-sectional analysis of a data derived from national demographic and family health survey administered by Peru’s national institute of statistics and information, the data input and possibly analysis may not be questionable. However multiple investigators involved in data collection without regular supervision leaves considerable scope for methodological errors in the acquisition of the data. The authors though have admitted about this possibility in the section on limitation of the study.

**Covariate section**:- Most of the covariant like age strata , gender, core behavior, socio-economic status, including education, income, rural, urban are factored in. However varying dietary habits, and occupation important covariant for hypertension need to be factored into. Rural to urban migration also plays important role in hypertension through life style changes.

**Result Section**:- The primary objective of this study is evaluation of association between high altitude and subtypes of hypertension. To my knowledge there is no such specific study on the subtypes of hypertension at high altitude. However most of the previous studies seem to agree with their findings that prevalence of hypertension at high altitude is lower than that at lower altitude at least up-to age 70 years. What should have been evaluated in this study is the trajectory of blood pressure at high altitude beyond 70 years (70-80 years and ≥ 80 years.) A conspicuous observation in the Indian Himalayan population is that up-to age 70 years, altitude do-not play a significant role in prevalence of hypertension. However there is a steep rise in both systolic and diastolic blood pressure after 70 years. Probably it implies an accelerated aging after 70 years as it happens with chronic mountain sickness (CMS) prevalence with advancing age. (reference1). The negative association of ISH with altitude and positive association with IDH as seen in this study is in agreement with previous studies at high altitude. (K.Otsuka, T Norboo etal 2005 Reference 2).

**Interpretation of results:-** ISH is mainly caused by increased stiffness and loss of dispensability of large elastic arteries due to aging as postulated by authors is substantiated by other investigators. (K Otsuka and T.Norboo et al 2005 reference 3.) K Otsuka et al showed an extreme increase in Cardio-Ankle –Vascular Index (CAVI) with SDH in subjects with advanced age at high altitude which indicates increasing stiffness and loss of dispensability of large elastic arteries.

Statistic Part:- I regret my inability to evaluate this part and request editorial group to let this part be reviewed by an expert biostatistician.

The study is an original research, though based on secondary cross sectional analysis data. This study on subtypes of hypertension (ISH, IDH and SDH) has not been published elsewhere to my knowledge. The methodology of statistical analysis are described in detail, though I admit my inability to evaluate the data and leave it to the editorial staff to decide about it. Conclusions are presented in an appropriate fashion and is in conformity with the objective. The article is presented in intelligible fashion and is written in Standard English.

Reference:-

1) Tsering Norboo, Tsering Stobdan, Norboo Tsering, Norboo Angchuk, Phunsog Tsering, Iqbal Ahmed, and Tsewang Chorol, et al. Prevalence of hypertension at High Altitude: Cross sectional survey in Ladakh, Northern India 2007-2011.

2) K Otsuka, T. Norboo et al. “ Effect of aging in blood pressure in Leh, Ladakh, a high altitude (3524m) community by comparison with a Japanese town” ELSEVIER; Biomedicine and pharmacotherapy,. 59(2005)554-557.

3) K Otsuka , T.Norboo et al. ‘Chroneocological health watch of arterial stiffness and neuro-cardio- pulmonary function in elderly community at high altitude(3524m). ELSEVIER; Biomedicine and pharmacotherapy. 59 (20050558-567).
