# Supplementary material for: Hypertension subtypes at high altitude in Peru: Analysis of the Demographic and Family Health Survey 2016–2019
Source: PLoS One. 2024 Apr 12;19(4):e0300457. doi: 10.1371/journal.pone.0300457 (PMC11014732; doi:10.1371/journal.pone.0300457)
Supplement: Attachment — Submitted filename: Response to Reviewers.docx [file pone.0300457.s003.docx]

**Hypertension subtypes at high altitude in Peru: Analysis of the Demographic and Family Health Survey 2016-2019**

**Reviewer #1:**

**Comment:** Introduction section: While few references have been mentioned regarding status of hypertension in high altitude regions of the world like south America, Nepal and Tibet, conspicuously missing is the reference on this subject in the Trans-Himalayan high altitude population of Indian part of Himalaya in Ladakh one of the highest inhabited regions in the Northernmost part of India. (BMJ Open 2015.) Reference 1. This study mentioned above concludes that like everywhere else in the world, hypertension prevalence in a high altitude population has multifactorial etiology. It shows that age, gender, socio-economic factors, culture, race and changing life style play a big role with the effect of high altitude itself on the prevalence of hypertension.

**Response:** Thank you very much for the comment. We added this information in the introduction (line 96 with reference No. 17, of the version with track changes).

**Comment:** Covariate section: Most of the covariant like age strata, gender, core behavior, socio-economic status, including education, income, rural, urban are factored in. However varying dietary habits, and occupation important covariant for hypertension need to be factored into. Rural to urban migration also plays important role in hypertension through life style changes.

**Response:** Thank you very much for observation. We also consider that it would be important to introduce these covariates in the analysis, however the survey does not have objective information on these variables. We selected covariates according to what was reported in previous studies and their availability in the survey.

**Comment:** Result Section: However most of the previous studies seem to agree with their findings that prevalence of hypertension at high altitude is lower than that at lower altitude at least up-to age 70 years. What should have been evaluated in this study is the trajectory of blood pressure at high altitude beyond 70 years (70-80 years and ≥ 80 years.) A conspicuous observation in the Indian Himalayan population is that up-to age 70 years, altitude do-not play a significant role in prevalence of hypertension. However, there is a steep rise in both systolic and diastolic blood pressure after 70 years. Probably it implies an accelerated aging after 70 years as it happens with chronic mountain sickness (CMS) prevalence with advancing age. (reference1). The negative association of ISH with altitude and positive association with IDH as seen in this study is in agreement with previous studies at high altitude. (K.Otsuka, T Norboo etal 2005 Reference 2).

**Response:** Thank you very much for your comment. For the age covariate, we used the cutoffs described in the “2020 International Society of Hypertension Global Hypertension Practice Guidelines.” Likewise, we added and discussed reference No. 2 in the discussion section (line 332, Revised Manuscript with Track Changes).

**Reviewer #2:**

**Comment:** Summary Section: Please include a subtitle for 'Background' to provide a clearer structure.

**Response:** We added the "Background" section in the summary.

**Comment:** Introduction Updates: Update the data on global hypertension deaths, preferably using statistics from around 2020.

**Response:** Thank you very much for your suggestion. We have updated the data.

**Comment:** Introduction Updates: Clarify the increase in the prevalence of hypertension in Peru. Include specific figures or percentages to quantify this increase.

**Response:** Thank you very much for your comment. We added the percentages (line 72 of the introduction section [Revised Manuscript with Track Changes]).

**Comment:** Introduction Updates: Incorporate data on the epidemiology of hypertension in Peru to provide context and relevance to your study.

**Response:** Thank you very much for your comment. We added the suggested information (lines 73 - 77 [Revised Manuscript with Track Changes]).

**Comment:** Add a 'Settings' section describing Peru's geographical divisions and altitude ranges.

**Response:** We added the “Settings” section along with the “Study Design and Data Source” section, aiming to provide better understanding to the reader.

**Comment:** Divide the 'Selection Criteria' into 'Inclusion Criteria' and 'Exclusion Criteria'.

**Response:** We carried out the suggested division.

**Comment:** Introduce a 'Bias' section explaining measures taken to minimize potential biases.

**Response:** Thanks a lot for the suggestion. We added the "Bias" section, and it is located on line 172 (Revised Manuscript with Track Changes).

**Comment:** Clarify the rationale behind the chosen altitude classification, especially in comparison to the International Society of Mountain Medicine’s classification.

**Response:** We thank the reviewer for highlighting the issue of high altitude classification. We have chosen 2500 meters above sea level (masl) as the cutoff for defining high altitude, as this is a widely recognized and practical benchmark in existing literature for studying the impact of high altitude on health. Furthermore, both the consensus on acute and chronic mountain sickness by the International Society of Mountain Medicine (ISMM) identify these conditions as occurring above 2500 masl (Roach RC et al., High Alt Med Biol 2018; Beall, Annu Rev Anthropol 2014). To clarify this, we have added the relevant references in the 'Variables: Exposure' section. Additionally, we conducted sensitivity analyses using other cutoff points (<500, 500-2,499, 2,500-3,499, ≥3,500 masl), as employed in a previous study with national data (Bernabe-Ortiz 2022, Glob Heart).

**Comment:** Results Consistency: Ensure that the altitude ranges discussed in the methodology align with those mentioned in the results.

**Response:** The results obtained with the cut-off point of 2500 masl are found in the manuscript, while the result of the sensitivity analyzes with other cut-off points (<500, 500-2,499, 2,500-3,499, ≥3,500 masl) are found in the file S1_File (supplementary material).

**Comment:** Figure Quality: Improve the resolution and clarity of Figures 1 and 2.

**Response:** We improved the quality of both figures.

**Comment:** Financial Disclosure: Relocate the 'Financial Disclosure Statement' to precede the reference list.

**Response:** Done.
